# Hypoalbuminemia, but not derived neutrophil to lymphocyte ratio (dNLR), predicts overall survival in neuroendocrine tumours undergoing peptide receptor radionuclide therapy: A retrospective, cohort study of 557 patients

**DOI:** 10.1111/jne.13379

**Published:** 2024-03-13

**Authors:** Dimitrios Papantoniou, Katarzyna Fröss‐Baron, Ulrike Garske‐Román, Anders Sundin, Espen Thiis‐Evensen, Malin Grönberg, Staffan Welin, Eva Tiensuu Janson

**Affiliations:** ^1^ Department of Medical Sciences, Endocrine Oncology Uppsala University Uppsala Sweden; ^2^ Department of Oncology Ryhov County Hospital Jönköping Sweden; ^3^ Department of Immunology, Genetics and Pathology Uppsala University Uppsala Sweden; ^4^ Department of Surgical Sciences, Radiology & Molecular Imaging Uppsala University Uppsala Sweden; ^5^ Department of Transplantation Medicine Oslo University Hospital, Rikshospitalet Oslo Norway

**Keywords:** CRP, dNLR, hypoalbuminemia, inflammatory markers, neuroendocrine tumour

## Abstract

Several inflammation scores have shown association with survival outcomes for patients with neuroendocrine tumours (NET) treated with peptide receptor radionuclide therapy (PRRT). However, whether these scores add value to established prognostic factors remains unknown. In this retrospective, cohort study of 557 NET patients undergoing PRRT in a tertiary referral centre from 2005 to 2015, we examined inflammatory markers and scores previously associated with cancer outcomes, using Cox proportional hazard models and Akaike's information criterion. Lower albumin (hazard ratio [95% confidence interval], .91 [.87–.95] per unit), as well as higher C‐reactive protein (CRP; 1.02 [1.01–1.02]), Glasgow Prognostic Score (GPS; 1 vs. 0: 1.67 [1.14–2.44], 2 vs. 0 3.60 [2.24–5.79]), CRP/albumin ratio (1.84 [1.43–2.37]) and platelet count (Plt) × CRP, but not white blood cell, neutrophil and thrombocyte counts or derived neutrophil to lymphocyte ratio (dNLR), were associated with shorter median overall survival (OS) in an adjusted analysis. The addition of parameters based on albumin and CRP, but not dNLR, to a base model including age, chromogranin A, the cell proliferation marker Ki‐67, performance status, tumour site and previous treatments improved the predictive accuracy of the base model. In an exploratory analysis of patients with available erythrocyte sedimentation rate (ESR) and CRP, ESR emerged as the most powerful predictor. When added to a prognostic model for OS in NET patients treated with PRRT, most inflammation scores further improved the model. Albumin was the single marker adding most value to the set of established prognostic markers, whereas dNLR did not seem to improve the model's prognostic ability.

## Graphical abstract

The addition of most established inflammation scores, but not dNLR, improved a prognostic model including age, chromogranin A, Ki‐67, performance status, tumour site and previous treatments in NET patients treated with PRRT. Albumin was the single marker that mostly reduced the base model's prediction error, as indicated by the lower Akaike's information criterion (AIC). Glasgow prognostic score (GPS), a composite score combining C‐reactive protein (CRP) and albumin values, marginally further improved the model.

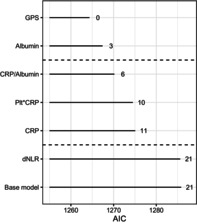

## 1 INTRODUCTION

Neuroendocrine tumours (NET) represent a rare group of neoplasms originating from neuroendocrine cells located in various organs. They are often indolent tumours with relatively long survival even when they have metastasized.^1^ The majority of NET express somatostatin receptors, which can be targeted with peptide receptor radionuclide therapy (PRRT). ^177^Lu‐DOTATATE has been used for PRRT of locally advanced or metastatic, unresectable NET and has been shown in a randomized trial to prolong progression‐free survival of small intestinal NET (SI‐NET) compared with high‐dose somatostatin analogues (SSA).^2^ Several factors, including baseline chromogranin A (CgA),^3^ age,^4^ prior treatments,^5^, ^6^ tumour proliferation index and Eastern cooperative oncology group performance status (PS)^5^ have been shown to be prognostic factors for overall survival (OS). There is, however, considerable heterogeneity in treatment response and new predictive and prognostic markers, which may offer added value to the established factors, are needed.

Cancer‐associated inflammation may be associated with treatment response in various cancers. Mono‐analytes including plasma levels of acute‐phase proteins, such as C‐reactive protein (CRP)^7^ and albumin,^8^ and the cellular components of the inflammatory response, such as lymphocytes and platelets,^9^, ^10^ have prognostic value in multiple cancer types. Also, several composite markers of inflammatory response have been developed. These include the Glasgow Prognostic Score (GPS), based on the presence of abnormal CRP and albumin levels,^11^ neutrophil to lymphocyte ratio (NLR),^12^ derived NLR (dNLR),^13^ platelet to lymphocyte ratio,^14^ CRP to albumin ratio^15^ and other high‐order combinations of blood tests. Meta‐analyses have evaluated their efficacy in various tumours. It is, however, unclear which score has the highest prognostic value.

Whether systemic inflammatory response plays an important role in NET is debated. Inflammatory markers have been investigated in small patient cohorts, showing some evidence of association with PRRT outcomes.^16^, ^17^, ^18^, ^19^, ^20^, ^21^, ^22^ However, no clear correlation was shown in one larger study.^23^ It is thus still uncertain whether inflammation‐based scores can be used to predict response to PRRT. Additionally, there is no evidence as to which inflammatory score has the highest prognostic value. Furthermore, when a new biomarker is evaluated, it is important to show whether it offers additional information compared with already established prognostic factors.^24^

The aim of this study was to compare previously identified inflammation markers and scores in a large cohort of NET patients treated with PRRT and to examine whether they provided added value to a set of established prognostic factors.

## 2 MATERIALS AND METHODS

### 2.1 Patients

This retrospective cohort study included 557 consecutive patients with metastatic inoperable NET treated with PRRT from 2005 to 2015 at Uppsala University Hospital, a tertiary referral centre. Patients were retrieved from a prospective internal database and additional data, including baseline inflammatory laboratory tests, were collected retrospectively. Inclusion criteria have previously been described.^6^, ^25^ In short, patients with progressive metastatic NET, with adequate organ function and adequate tumour somatostatin receptor expression were eligible for inclusion. Chemotherapy, targeted therapies and interferon were stopped at least 1 month prior to PRRT. PRRT was administered according to previously published procedures.^25^ Briefly, 7.4 GBq ^177^Lu‐DOTATATE was administered with 6‐ to 8‐week intended intervals. The vast majority of patients were treated according to a dosimetry‐guided protocol (EudraCT nr 2009–012260‐14). Treatment continued until an accumulated dose of 23 Gy to the kidneys or 2 Gy to the bone marrow or other reasons to stop therapy occurred. Selected patients progressing after initial favourable tumour response were eligible for additional salvage therapy with up to 40 Gy accumulated absorbed dose to the kidneys.

### 2.2 Follow‐up and data collection

Medical records were retrospectively evaluated. All patients had routine blood tests on the day of hospitalization prior to treatment start. Erythrocyte sedimentation rate (ESR) was only collected for 200 patients, treated during the early years of the study and is therefore not included in the main analysis. CRP was missing for 210 patients. All other blood parameters were collected systematically throughout the study period, as detailed below. The proliferation marker Ki‐67 was extracted from pathology records. In case of multiple biopsies, the highest Ki‐67 value before PRRT start was used. Survival status was censored on 28 May 2023, or on the date for last available follow‐up.

### 2.3 Inflammation scores

The following inflammation‐based mono‐analytes and composite scores previously used in NET studies were evaluated: Absolute white blood cell (WBC), neutrophil and thrombocyte counts, CRP, albumin, dNLR, GPS, CRP to albumin ratio and platelet count (Plt) × CRP. The dNLR was defined as neutrophil/(WBC−neutrophil). Most NET studies use dNLR/NLR cutoffs internally tailored to fit the data. Those range from 1.7 to 2.6, with dNLR lingering on the lower side.^16^, ^17^, ^18^ For this study, an arbitrary cutoff of 2 was chosen ad hoc to distinguish high and low dNLR values. GPS was calculated from CRP and albumin, assigning one point each to CRP >10 mg/L or albumin <35 g/L. CRP to albumin ratio was calculated as CRP/albumin, whereas Plt × CRP was calculated by multiplying platelet and CRP counts.

### 2.4 Statistical methods

The primary endpoint was OS, defined as time from treatment start to death from any cause. OS was analysed using the Kaplan–Meier method, and between‐group differences were evaluated using a log‐rank test. Hazard ratios (HRs) and confidence intervals (CIs) were estimated from the Cox proportional hazards model applied to the whole cohort (*n* = 557). CRP and albumin were analysed as both continuous and dichotomous variables, with cutoffs of 10 and 35 mg/L as used in GPS. As this work is perceived as confirmatory for the main set of previously reported predictors, a Bonferroni correction was used; accounting for 12 parameters, a *p*‐value <.004 was considered significant. Additional markers were exploratory.

In the group of patients for whom all laboratory parameters were available (CRP cohort, *n* = 347), Akaike's information criterion (AIC) was used to estimate goodness‐of‐fit for each inflammation score. AIC is an estimator of prediction error and allows for the comparison of statistical models developed on a specific (same) dataset. When comparing two models, the absolute difference of AIC (∆_AIC_) represents the information loss when not using the best model (the model with lower AIC). A ∆_AIC_ ≤ 2 is generally considered insignificant, meaning that the two models are comparable. When 4 ≤ ∆_AIC_ ≤ 7, there is less support for the model with higher AIC, whereas if ∆_AIC_ > 10, the model with higher AIC has considerably worse predictive accuracy.^26^ An additional exploratory analysis was conducted in the patients in whom ESR had been collected (ESR cohort, *n* = 199, Supporting Information).

Statistical analysis was performed with R version 4.1.2 (R Foundation for Statistical Computing, Vienna, Austria, RRID: SCR_001905) and AIC was calculated with the AICcmodavg package (RRID: SCR_023299) version 2.3. All tests were two‐sided.

## 3 RESULTS

### 3.1 Patient demographics

Complete laboratory data were available for 347 of 557 patients. Median age was 63 years (interquartile range, 55–69). The most frequent tumour location was small intestine (51%), followed by pancreas (25%) (Table 1). One third of the patients had high dNLR and low albumin, and one fifth had high CRP (Table 2). The majority had grade 1 (29%) and grade 2 tumours (66%). Ki‐67 was missing in approximately 15% of the patients. During a median follow‐up time of 37 months, 366 patients (66%) had died.

**TABLE 1:** Baseline patient characteristics.

	All	All inflammation markers
	*N* = 557	*N* = 347
Age	63 (55–69)	62 (52–68)
Performance status		
0	341 (62%)	207 (61%)
1	153 (28%)	97 (29%)
≥2	53 (10%)	36 (11%)
Tumour location		
Small intestine	281 (51%)	157 (46%)
Pancreas	139 (25%)	92 (27%)
Other	131 (24%)	93 (27%)
Chromogranin A (×ULN)	9 (2–28)	8 (2–29)
Previous lines of therapy except SSA		
0	185 (34%)	107 (31%)
1	255 (46%)	159 (46%)
≥2	111 (20%)	77 (22%)
Ki‐67	6 (2–12)	5 (2–11)
Grade		
1	136 (29%)	86 (30%)
2	308 (66%)	188 (66%)
3	26 (6%)	9 (3%)

*Note*: Values are median (interquartile range) or *N* (%).

Abbreviations: SSA, somatostatin analogues; ULN, upper limit of normal.

**TABLE 2:** Association between inflammation markers and OS.

OS		*n*	HR (unadjusted)	*p*	HR (adjusted)^a^	*p*
CRP		351	1.02 (1.02–1.03)	**<.001**	1.02 (1.01–1.02)	**<.001**
Albumin		556	.89 (.87–.91)	**<.001**	.91 (.87–.95)	**<.001**
White blood cells		556	.99 (.94–1.03)	.555	.96 (.89–1.03)	.279
Neutrophils		555	1.05 (.98–1.12)	.136	1.00 (.91–1.10)	.989
Thrombocytes		556	1.00 (1.00–1.00)	.496	1.00 (1.00–1.00)	.906
Albumin, dich	Low	178	‐		‐	
	Normal	378	.43 (.35–.54)	**<.001**	.39 (.27–.55)	**<.001**
CRP, dich	Normal	279	‐		‐	
	Elevated	72	2.02 (1.47–2.79)	**<.001**	1.75 (1.18–2.59)	.005
dNLR		555	1.19 (1.08–1.31)	**<.001**	1.11 (.98–1.25)	.111
dNLR, dich	Low	364	‐		‐	
	High	191	1.44 (1.16–1.78)	**.001**	1.37 (.99–1.90)	.060
GPS	0	210	‐		‐	
	1	99	2.05 (1.51–2.77)	**<.001**	1.67 (1.14–2.44)	.008
	2	42	3.59 (2.40–5.36)	**<.001**	3.60 (2.24–5.79)	**<.001**
CRP/albumin ratio		351	2.27 (1.86–2.78)	**<.001**	1.84 (1.43–2.37)	**<.001**
(Plt × CRP)/1000		351	1.08 (1.05–1.10)	**<.001**	1.06 (1.03–1.08)	**<.001**

Abbreviations: CRP, C‐reactive protein; dNLR, derived neutrophil to lymphocyte ratio; GPS, Glasgow Prognostic Score; HR, hazard ratio; OS, overall survival; Plt, platelet count; SI‐NET, small intestinal neuroendocrine tumours.

^a^ Adjusted for age, ki‐67, logarithmically transformed normalized chromogranin A, number of previous treatment lines (0, 1, ≥2), performance status (0, 1, ≥2) at treatment start and stratified by tumour type (SI‐NET vs. pancreas vs. other). The whole dataset was used for unadjusted analyses, whereas adjusted analyses were performed on the patients with complete lab tests and Ki‐67 (*n* = 273). *p*‐values <.004 (in bold) were considered significant to adjust for multiple comparisons.

### 3.2 Relation between inflammation scores and survival

**FIGURE 1:**
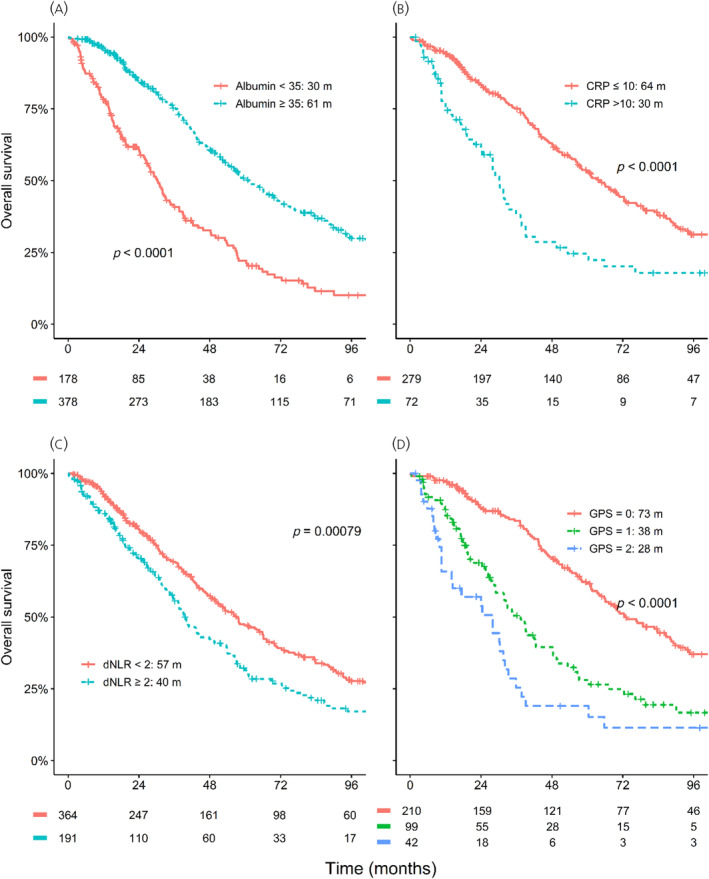
Overall survival by (A) albumin, (B) C‐reactive protein (CRP), (C) derived neutrophil to lymphocyte ratio (dNLR) and (D) Glasgow Prognostic Score (GPS).

Higher CRP, GPS, dNLR, CRP/albumin ratio and Plt × CRP, as well as lower albumin, were associated with shorter median OS in the unadjusted analysis. No relation between WBC, neutrophil or thrombocyte counts and OS could be established (Table 2 and Figure 1). In a subgroup analysis, albumin, CRP and GPS remained associated with OS in SI‐NET, pancreatic NET and NET of other origin. The direction of effect was similar for Grade 1 and Grade 2/3 tumours, but with the exception of CRP and GPS 2 versus 0, differences were significant only in higher grade tumours (Table S1).

CRP, albumin and most composite inflammation scores, but not dNLR, remained as independent prognostic factors for OS after adjusting for age, CgA, PS, Ki‐67, tumour site and number of previous treatments (Table 2). In subgroup analyses, dNLR was still not an independent prognosticator irrespective of NET type (adjusted HR [95% CI] for SI‐NET, pancreas NET or NET in other locations of 1.32 [.94–1.86], 1.17 [.66–2.07]) and 1.11 [.65–1.89], respectively, or Ki‐67 levels (<10%: 1.16 [.84–1.59], ≥10%: 1.24 [.83–1.85]).

### 3.3 Comparison of inflammation scores

**FIGURE 2:**
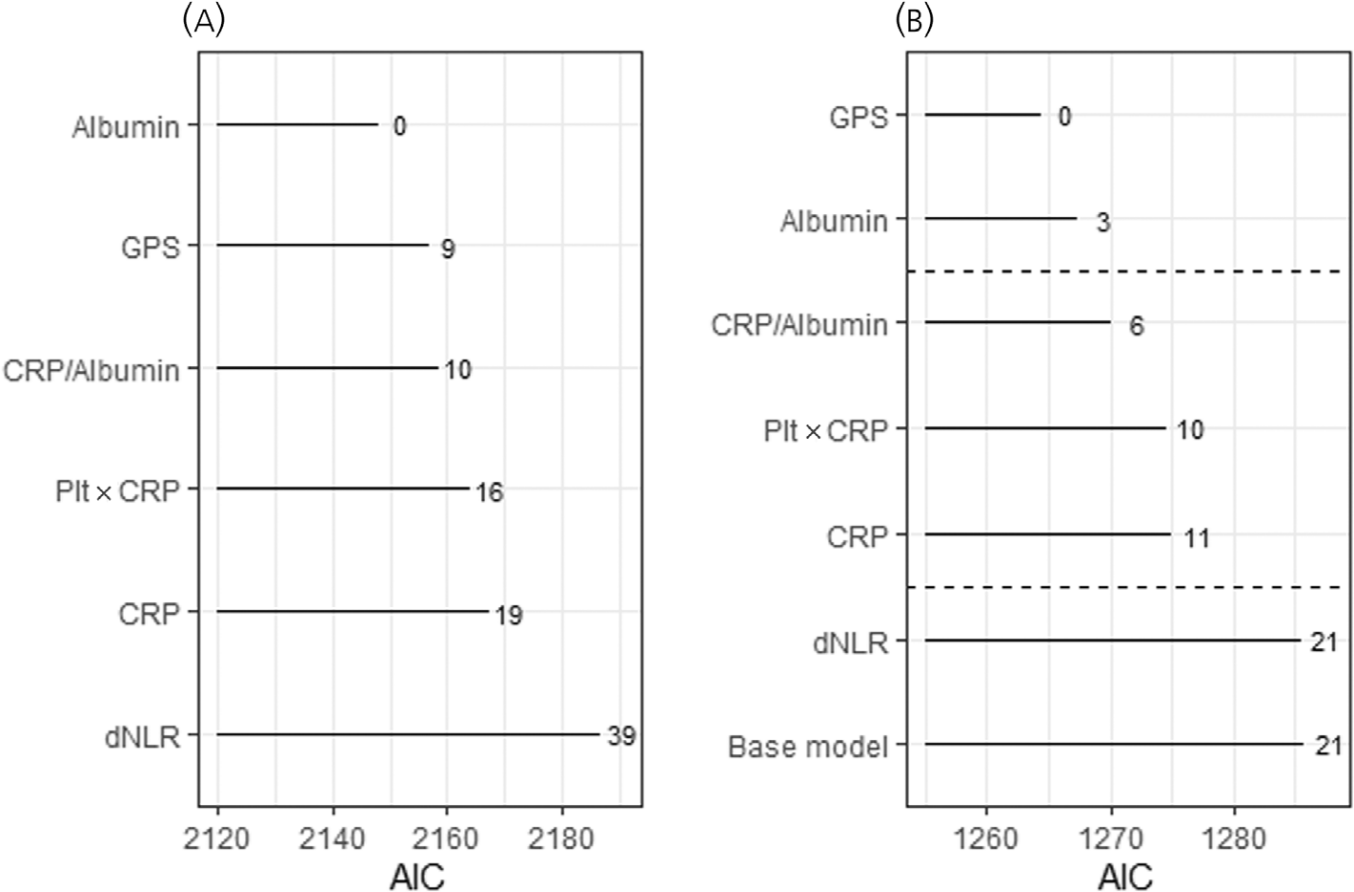
Predictive accuracies of unadjusted and adjusted models. (A) Unadjusted. (B) Added to the base model. While comparing two models, lower values signify lower prediction error (a more informative model), and thus a model that better fits the test data. Base model includes age, tumour type, Ki‐67, logarithmically transformed normalized Chromogranin A, number of previous treatment lines, excluding SSA and performance status at treatment start. All parameters but derived neutrophil to lymphocyte ratio (dNLR) improved the base model. Albumin was the single parameter that mostly reduced the base model's prediction error. Glasgow Prognostic Score (GPS), a composite score combining C‐reactive protein (CRP) and albumin values, marginally further improved the adjusted model. AIC, Akaike's information criterion; Plt, platelet count.

Models including parameters based on albumin and CRP outperformed those based on blood cell counts in unadjusted analysis. More importantly, all albumin and CRP‐based markers substantially improved the base model, as indicated by the substantially lower AIC (∆_AIC_ > 10), whereas no improvement was observed with dNLR (∆_AIC_ = 0 compared with base model) (Figure 2). AIC is a measure of the prediction error and can be used to compare two models derived from the same dataset; a lower AIC signifies a more accurate model. A difference of >4 is generally considered as some evidence, and >10 as strong evidence of a better prognostic ability.^26^ Albumin and its combination with CRP were the markers associated with lower prediction error, and thus could more accurately predict OS, in both unadjusted and nested models (Figure 2).

In an exploratory analysis including the subgroup of 199 patients with all available inflammatory markers, ESR was strongly associated with OS (adjusted HR per one unit increase [95% CI], 1.02 [1.01–1.03]; elevated vs. normal, 2.67 [1.59–4.49]). The univariable model based on ESR outperformed all other single‐parameter models, whereas a multivariable model based on albumin and ESR was the most informative model (Figure S1).

## 4 DISCUSSION

In this study of 557 NET patients treated with PRRT, examining inflammatory markers and prognostic scores previously associated with cancer outcomes, we showed that CRP and albumin‐based markers were independently associated with OS but could not detect an association for dNLR and other cellular inflammation‐based markers. Furthermore, hypoalbuminemia was the single most predictive marker showing the most added predictive value in a model derived from established prognostic factors.

A useful biomarker has to be reproducible, easy to measure, inexpensive and add prognostic value in terms of outcomes.^27^ As inflammation scores are based on routine blood tests, reproducibility and accessibility are excellent. Moreover, a new biomarker should increase the information provided by models derived from already known prognostic factors. This information can be assessed by model goodness‐of‐fit statistics, such as the AIC. We found that, with the exception of dNLR, all tested inflammatory parameters improved the prognostic ability of a base model consisting of age, CgA, previous treatment lines, tumour type and PS. Hypoalbuminemia provided the most added information, whereas CRP only marginally improved the models. Based on similar AIC values, both hypoalbuminemia and its combination with CRP, as expressed in the GPS, emerge as possible stratification factors for future clinical trials and prognostic research. According to the principle of parsimony, between two models fitting the data similarly well, the simpler model with fewer parameters is preferred. Pending validation in an external study, our study would thus suggest low albumin as the most useful prognostic factor.

**FIGURE 3:**
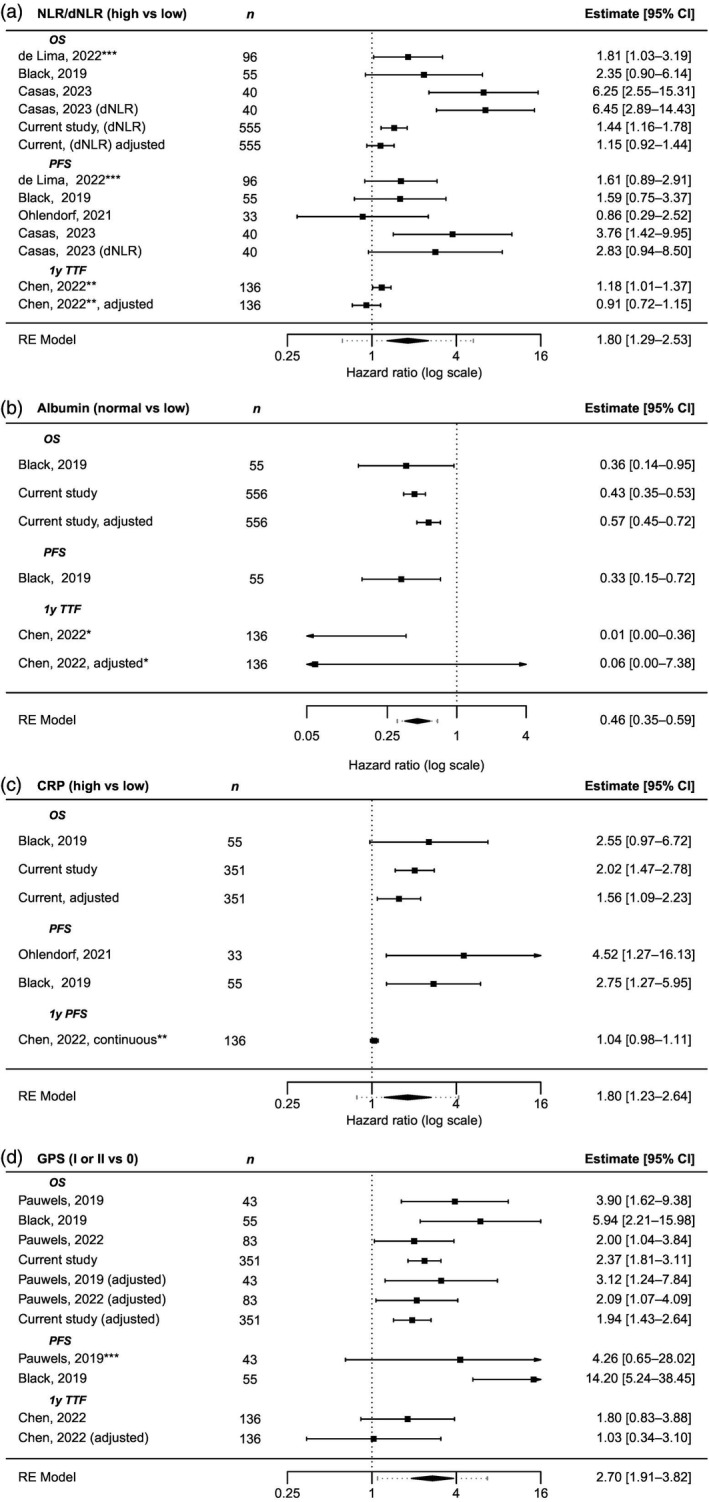
Association between survival outcomes and inflammatory parameters in the literature. Adjusted and unadjusted hazard ratios for overall survival (OS), progression‐free survival (PFS), and 1‐year treatment failure (1y TTF) for (A) NLR/derived neutrophil to lymphocyte ratio (dNLR), (B) albumin, (C) C‐reactive protein (CRP) and (D) Glasgow Prognostic Score (GPS). Hazard ratios (HRs) are unadjusted and variables are dichotomized, unless otherwise noted. HR (log scale). Higher values denote increasing risk of death or progression. HRs >1 denote worse prognosis. *Extreme predictions as only 6 patients with hypoalbuminemia; **continuous; ***estimated.

A recent umbrella review of 204 meta‐analyses of observational studies on neutrophil counts suggested an association between elevated NLR and poor outcomes across multiple cancer types, although the authors noted some limitations due to study quality and small‐study effects.^12^ Unexpectedly, dNLR and OS were unrelated in the present study. This held true irrespective of NET type or Ki‐67 levels. These results contrast with those of previous small retrospective studies (Figure 3A).^16^, ^17^, ^18^, ^20^ In addition to the limited number of patients, these earlier studies used custom cutoffs (either the median or a cutoff tailored to maximize HR within that sample), which do not necessarily generalize to other populations. Moreover, in our cohort, dNLR was prognostic in an unadjusted analysis (Figure 1C), but not after adjustment for potential confounders. Similarly, the only other study on >100 patients found that NLR did not retain its prognostic value for 1‐year treatment failure (1y TTF) in the adjusted analysis.^23^ The reason for this discrepancy is unclear. Possible explanations are that NET might not cause the same level of activation of cellular systemic inflammation as more aggressive cancer types or that neutrophil‐mediated cancer promotion does not have the same role in PRRT as in other systemic therapies.

Interestingly, we found that low albumin levels strongly associated with worse OS. One previous study reported that hypoalbuminemia is a negative prognostic factor for OS and PFS, whereas a larger study could not establish a relation with 1y TTF (Figure 3B). Hypoalbuminemia has been reported in up to 30%–50% in cancer patients and is more prevalent in late disease stages. Although hypoalbuminemia is likely to be multi‐factorial, albumin levels have been suggested to be a more potent indicator of inflammation than of low nutritional status.^28^ Indeed, in our study, there was a stronger correlation between hypoalbuminemia and inflammatory parameters, such as CRP (Pearson's *r* = −.45), than to body mass index (*r* = .16) or degree of hepatic involvement (point biserial correlation coefficient *r*_pb_ = .26). In an exploratory analysis, hypoalbuminemia remained an independent prognostic factor for OS after adjusting for metastatic burden in the liver (Figure S2). Hypoalbuminemia has been associated with surgical complications and morbidity,^29^ poor survival outcomes in multiple cancer types^8^ and higher toxicity of chemotherapy, targeted therapy and external radiotherapy.^30^, ^31^, ^32^

Unexpectedly, ESR was found to have higher predictive value than CRP in a subset of patients for whom both measurements were available. Unlike CRP, which is a direct marker of acute inflammatory reaction, ESR is an indirect marker of inflammation. ESR levels increase slower, remain elevated for longer periods of time and are less specific than CRP; thus CRP is preferred in most, but not all situations.^33^ Although a higher CRP has been associated with poor outcomes in multiple cancer types, including NET in some but not all studies (Figure 3C), evidence for ESR is scarce,^34^, ^35^, ^36^, ^37^ and the association between ESR and OS in patients treated with PRRT has not been studied previously. Our findings indicate a potential role of ESR for identifying patients who might respond poorly to PRRT. However, as this finding was merely based on a subset of patients treated during the initial years of our study, we consider it preliminary and in need of further validation.

As data on other treatments were limited, we could not determine whether inflammatory markers, apart from their prognostic significance, can also predict treatment effect specifically after PRRT. Interestingly, in a previously presented subset of 115 patients with both SSA and PRRT baseline data,^38^ there seemed to be association between inflammatory markers and survival for treatment with PRRT but not with SSA. This might signify either an intrinsic difference between the two treatment types or a more inflammatory disease status at later stages (Table S2).

Our study has several limitations. First, 210 patients were excluded from comparison models because of missing inflammatory parameters, mostly CRP, potentially introducing an unintended bias. However, in a real‐world retrospective study, parameter collection can be expected to differ over time. All comparisons were performed in subsets of patients for whom all parameters were available. Second, lymphocyte, eosinophil and monocyte counts were not routinely collected. Consequently, scores such as the albumin to monocyte ratio, or platelet to lymphocyte ratio could not be calculated. Third, we studied dNLR instead of the more frequently used NLR. As lack of information on lymphocytes is common in clinical trials, dNLR has been suggested to have similar prognostic value.^13^ The association between dNLR and survival outcomes has been shown in meta‐analyses in multiple tumour types.^39^, ^40^, ^41^ Indeed, the only study reporting on both dNLR and NLR in NET patients shows similar effect sizes.^16^ Fourth, inflammation markers and particularly ESR and CRP might be influenced by both the underlying inflammatory status and transient infections. Although minor infections cannot completely ruled out, patients included in the analysis did not have major conditions that would deem them clinically unfit for PRRT on the day of admission and blood sampling. Finally, as detailed information on metastatic status was missing in a third of the patients, we did not include this factor in the base model. However, all patients were metastatic at baseline, and more than 95% had liver metastases. In a separate analysis adding to the model information on bone and peritoneal disease, which have been associated with worse survival, albumin‐based markers were still strongly correlated to OS (data not shown). The main strength of our study is the size of the underlying cohort, which makes it the largest study examining inflammation markers in NET patients treated with PRRT. Additionally, to the best of our knowledge, it is the only study examining not only if inflammatory variables are independent predictors of OS but also whether they offer added value to established prognostic parameters.

In conclusion, we show that most established inflammation scores, but not cellular inflammation markers, correlate with OS in NET patients treated with PRRT, particularly for Grade 2/3 patients, and that hypoalbuminemia adds the highest prognostic value to a model composed of established prognostic factors. ESR emerges as a potential powerful biomarker, pending further validation. Our results strongly suggest that inflammation scores could be incorporated as stratification factors in future PRRT trials.

## AUTHOR CONTRIBUTIONS

**Dimitrios Papantoniou:** Conceptualization; data curation; formal analysis; investigation; visualization; writing – original draft; writing – review and editing. **Katarzyna Fröss‐Baron:** Conceptualization; investigation; writing – review and editing. **Ulrike Garske‐Román:** Conceptualization; investigation; writing – review and editing. **Anders Sundin:** Investigation; writing – review and editing. **Espen Thiis‐Evensen:** Investigation; writing – review and editing. **Malin Grönberg:** Investigation; writing – review and editing. **Staffan Welin:** Investigation; writing – review and editing. **Eva Tiensuu Janson:** Investigation; supervision; writing – original draft; writing – review and editing.

## FUNDING INFORMATION

This study was supported by the Swedish Cancer Society (grant number 200921) and Futurum—the Academy for Health and Care, Region Jönköping County.

## CONFLICT OF INTEREST STATEMENT

The authors declare no conflict of interest that could be perceived as prejudicing the impartiality of the research reported.

### PEER REVIEW

The peer review history for this article is available at https://www.webofscience.com/api/gateway/wos/peer-review/10.1111/jne.13379.

## ETHICS STATEMENT

All procedures within this study were in accordance with the 1964 Helsinki Declaration and its later amendments. Two hundred patients were included in a previous prospective study (EudraCT nr 2009–012260‐14, EPN 2009–320) after providing written informed consent. For the remaining patients the local ethics committee approved an amendment to the original application for retrospective review of medical records and data collection without the patients' informed consent.

## Supporting information


**Data S1.** Supporting Information.

## Data Availability

The data that support the findings of this study are available upon reasonable request from the corresponding author (Dimitrios Papantoniou). The data are not publicly available due to their containing information that could compromise the privacy of research participants.
