# Supplementary material for: Hypoalbuminemia, but not derived neutrophil to lymphocyte ratio (dNLR), predicts overall survival in neuroendocrine tumours undergoing peptide receptor radionuclide therapy: A retrospective, cohort study of 557 patients
Source: J Neuroendocrinol. 2024 Mar 13;37(3):e13379. doi: 10.1111/jne.13379 (PMC11919477; doi:10.1111/jne.13379)
Supplement: Supplementary file 1 — Data S1. Supporting Information. [file JNE-37-e13379-s001.docx]

#### Supplementary Fig. 1


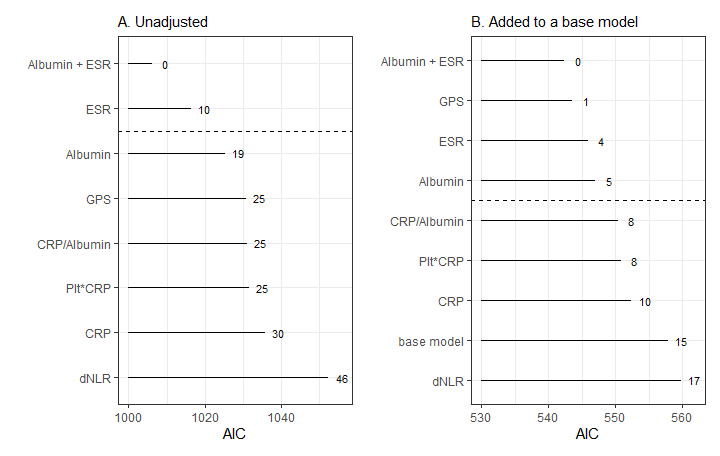


In a subset of 199 patients with both ESR and CRP, ESR seemed to have similar prognostic value to albumin in multivariable, and outperform all other inflammatory biomarkers in univariable analysis. Adding ESR to albumin moderately improved the albumin+base model (∆_AIC_ =5). ESR: erythrocyte sedimentation rate, dNLR: derived neutrophil to lymphocyte ratio, GPS: Glasgow prognostic score, CRP: C-reactive protein, PLT: platelets, AIC: Akaike information criterion.

##

#### Supplement Fig. 2


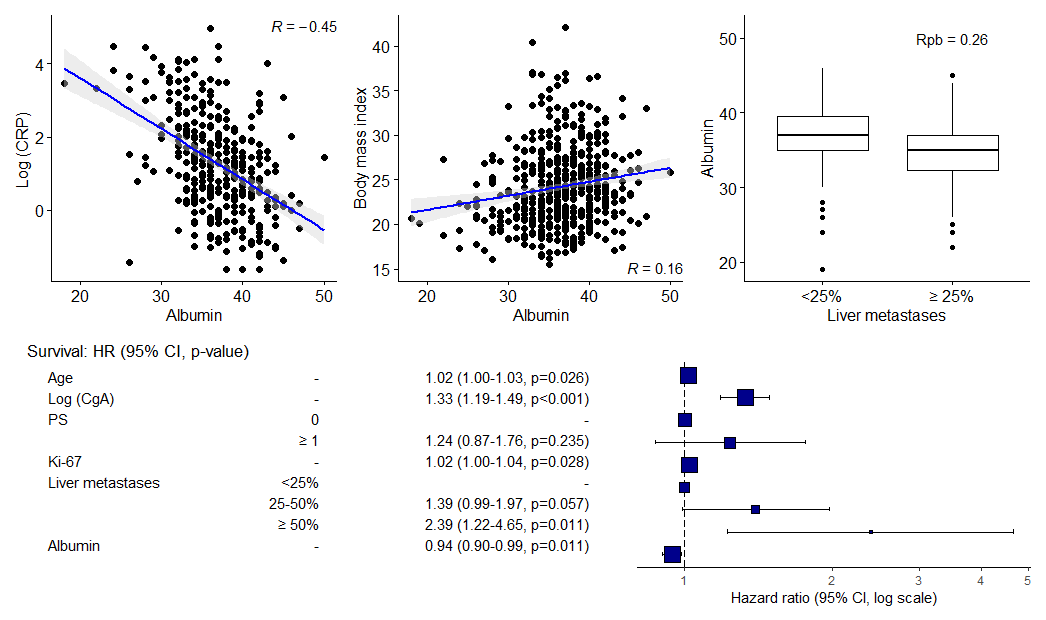


Hypoalbuminemia is likely to be multifactorial in patients with advanced cancer. Inflammation markers such as CRP were mostly associated with albumin levels (Pearson’s r=-0.45), followed by liver tumour burden (point biserial correlation coefficient r_pb_ = 0.26) and nutrition status, as expressed by body-mass index (r=0.16). In an exploratory analysis of the 261 patients with available liver imaging data, hypoalbuminemia remained an independent prognostic factor for overall survival after adjusting for the hepatic metastatic burden. HR: hazard ratio, PS: performance status, CgA: Chromogranin A. Values to the right of the dashed line indicated increased risk of death.

#### Supplement Table 1. Unadjusted analysis of the association between OS and inflammation markers by A. tumour type B. Grade

| A | SI-NET | | | | Pancreas | | | Other | | |
| --- | --- | --- | --- | --- | --- | --- | --- | --- | --- | --- |
|  | | *HR* | *p* | *N* | *HR* | *p* | *N* | *HR* | *p* | *N* |
| Albumin | | 0.89 [0.86;0.93] | <0.001 | 280 | 0.89 [0.84;0.94] | <0.001 | 139 | 0.87 [0.82;0.91] | <0.001 | 131 |
| CRP | | 1.03 [1.02;1.04] | <0.001 | 158 | 1.04 [1.02;1.06] | 0.001 | 94 | 1.02 [1.01;1.03] | <0.001 | 94 |
| dNLR | | 1.14 [1.00;1.31] | 0.052 | 280 | 1.24 [1.05;1.48] | 0.012 | 139 | 1.19 [0.96;1.46] | 0.105 | 130 |
| GPS | |  |  | 158 |  |  | 94 |  |  | 94 |
| 0 | | Ref. | Ref. |  | Ref. | Ref. |  | Ref. | Ref. |  |
| 1 | | 1.68 [1.07;2.63] | 0.025 |  | 1.98 [1.04;3.79] | 0.039 |  | 3.21 [1.79;5.75] | <0.001 |  |
| 2 | | 3.72 [2.19;6.34] | <0.001 |  | 2.33 [1.00;5.43] | 0.050 |  | 10.1 [3.75;27.1] | <0.001 |  |
|  | |  |  |  |  |  |  |  |  |  |
|  | |  |  |  |  |  |  |  |  |  |
| B | | Grade 1 | | | Grade 2/3 | | |  |  |  |
|  | | *HR* | *p* | *N* | *HR* | *p* | *N* |  |  |  |
| Albumin | | 0.97 [0.92;1.03] | 0.291 | 136 | 0.87 [0.84;0.90] | <0.001 | 334 |  |  |  |
| CRP | | 1.03 [1.00;1.05] | 0.019 | 87 | 1.02 [1.01;1.03] | <0.001 | 200 |  |  |  |
| dNLR | | 1.14 [0.95;1.37] | 0.145 | 136 | 1.25 [1.11;1.40] | <0.001 | 333 |  |  |  |
| GPS | |  |  | 87 |  |  | 200 |  |  |  |
| 0 | | Ref. | Ref. |  | Ref. | Ref. |  |  |  |  |
| 1 | | 0.84 [0.41;1.74] | 0.639 |  | 2.77 [1.85;4.15] | <0.001 |  |  |  |  |
| 2 | | 7.06 [2.26;22.1] | 0.001 |  | 5.17 [3.18;8.39] | <0.001 |  |  |  |  |

A. Inflammation markers appear prognostic of OS irrespective of tumour primary site in unadjusted analysis. B. The direction of effect is consistent among all grades, but differences are not significant in lower grade tumours. Due to the small number of patients in some subgroups, we did not perform adjusted analyses. As only 26 patients had grade 3 tumours, grade 2 and 3 are grouped together. HR: Hazard ratio, CRP: C-reactive protein, dNLR: derived neutrophil-to-lymphocyte ratio, GPS: Glasgow prognostic score.

#### Table 2. Prognostic significance for OS of selected inflammation markers in 115 siNET patients treated with both SSA and PRRT

|  | *HR* | *p* | *N* | *HR* | *p.ratio* | *N* |
| --- | --- | --- | --- | --- | --- | --- |
| CRP | 1.02 [0.98;1.07] | 0.278 | 35 | 1.04 [1.02;1.06] | **<0.001** | 67 |
| Albumin | 0.96 [0.91;1.01] | 0.120 | 65 | 0.88 [0.83;0.94] | **<0.001** | 115 |
| dNLR | 1.13 [0.86;1.49] | 0.388 | 60 | 1.17 [1.01;1.35] | 0.033 | 113 |
| GPS: |  |  | 32 |  |  | 67 |
| 0 | Ref. | Ref. |  | Ref. | Ref. |  |
| 1 | 2.63 [0.74;9.31] | 0.135 |  | 3.75 [1.94;7.25] | **<0.001** |  |
| 2 | 1.26 [0.16;9.61] | 0.826 |  | 3.76 [1.52;9.27] | **0.004** |  |

We evaluated baseline CRP, albumin, derived neutrophil to lymphocyte ratio (dNLR) and Glasgow prognostic score (GPS) as prognostic factors for overall survival (OS) in 115 patients with available data for treatment with both SSA and PRRT. OS was not associated with any inflammatory marker at the time of SSA initiation. Because of many missing values in the case of treatment with SSA, we could not adjust for confounders. The differences observed might be due to treatment given (SSA vs PRRT) or because tumours might become more inflammatory as the disease progresses (SSA was typically a 1^st^ line treatment whereas PRRT a 2^nd^/3^rd^ line treatment). SSA: somatostatin analogues, PRRT: peptide receptor radionuclide therapy. Applying a bonferroni correction, p-values <0.0125 are considered significant.
